# Thymidine Kinase 2 Deficiency-Induced Mitochondrial DNA Depletion Causes Abnormal Development of Adipose Tissues and Adipokine Levels in Mice

**DOI:** 10.1371/journal.pone.0029691

**Published:** 2011-12-27

**Authors:** Joan Villarroya, Beatriz Dorado, Maya R. Vilà, Elena Garcia-Arumí, Pere Domingo, Marta Giralt, Michio Hirano, Francesc Villarroya

**Affiliations:** 1 Departament de Bioquímica i Biologia Molecular, and Institut de Biomedicina (IBUB), Universitat de Barcelona, Barcelona, Spain; 2 Institut de Recerca de l′Hospital de la Santa Creu i Sant Pau, Barcelona, Spain; 3 Centro de Investigación Biomédica en Red Fisiopatología de la Obesidad y Nutrición (CIBERObn), ISCIII, Santiago de Compostela, Spain; 4 Department of Neurology, Columbia University Medical Center, New York, New York, United States of America; 5 Institut de Recerca de l′Hospital Universitari de la Vall d'Hebron, Barcelona, Spain; 6 Centro de Investigación Biomédica en Red de Enfermedades Raras (CIBERER), ISCIII, Valencia, Spain; Louisiana State University, Pennington Biomedical Research Center, United States of America

## Abstract

Mammal adipose tissues require mitochondrial activity for proper development and differentiation. The components of the mitochondrial respiratory chain/oxidative phosphorylation system (OXPHOS) are encoded by both mitochondrial and nuclear genomes. The maintenance of mitochondrial DNA (mtDNA) is a key element for a functional mitochondrial oxidative activity in mammalian cells. To ascertain the role of mtDNA levels in adipose tissue, we have analyzed the alterations in white (WAT) and brown (BAT) adipose tissues in thymidine kinase 2 (*Tk2*) H126N knockin mice, a model of TK2 deficiency-induced mtDNA depletion. We observed respectively severe and moderate mtDNA depletion in TK2-deficient BAT and WAT, showing both tissues moderate hypotrophy and reduced fat accumulation. Electron microscopy revealed altered mitochondrial morphology in brown but not in white adipocytes from TK2-deficient mice. Although significant reduction in mtDNA-encoded transcripts was observed both in WAT and BAT, protein levels from distinct OXPHOS complexes were significantly reduced only in TK2-deficient BAT. Accordingly, the activity of cytochrome c oxidase was significantly lowered only in BAT from TK2-deficient mice. The analysis of transcripts encoding up to fourteen components of specific adipose tissue functions revealed that, in both TK2-deficient WAT and BAT, there was a consistent reduction of thermogenesis related gene expression and a severe reduction in leptin mRNA. Reduced levels of resistin mRNA were found in BAT from TK2-deficient mice. Analysis of serum indicated a dramatic reduction in circulating levels of leptin and resistin. In summary, our present study establishes that mtDNA depletion leads to a moderate impairment in mitochondrial respiratory function, especially in BAT, causes substantial alterations in WAT and BAT development, and has a profound impact in the endocrine properties of adipose tissues.

## Introduction

Mitochondrial function is essential for the development and differentiation of the two types of adipose tissue in mammals, brown adipose tissue (BAT) and white adipose tissue (WAT). It has long been recognized that mitochondrial activity is a major component of the differentiated phenotype of BAT, as it provides the oxidative activity required for the thermogenic function of this type of adipose tissue. In contrast, the relevance of mitochondria to WAT function was often neglected. Several recent reports, however, have established the importance of mitochondrial biogenesis as part of the differentiation of the white adipose cell and the acquisition of its specific metabolic features [1], [2], [3], [4].

The molecular machinery of the mammalian mitochondrial respiratory chain and oxidative phosphorylation (OXPHOS) system has the unique feature of being encoded by two distinct genomes, the nuclear and the mitochondrial genome. The mitochondrial DNA (mtDNA) encodes 13 subunits of OXPHOS complexes I, III, IV and V that are essential for the enzymatic function of the OXPHOS system. Thus, coordinate expression of the OXPHOS proteins encoded by the nuclear DNA (nDNA) and mtDNA genes is required for functional mitochondrial oxidative activity [5], [6].

Alterations in mtDNA, from pathogenic point mutations to abnormally low amounts of mtDNA (mtDNA depletion), have been reported to cause multiple pathologies. These usually manifest as neurodegenerative and neuromuscular diseases, but there are also indications that altered mtDNA may lead to disturbances in adipose tissue [7]. Several reports have indicated associations of mtDNA polymorphic mutated forms with obesity and type II diabetes [8], [9], [10], [11]. However, the most-reported relationship between adipose tissue alterations and mtDNA depletion has been the lipodystrophy syndrome that appears in HIV-1-infected patients undergoing antiretroviral treatment. In these patients, the combination of viral infection and secondary effects of the treatment with inhibitors of the viral reverse transcriptase leads to abnormal mtDNA replication and a profound depletion of mtDNA levels in several tissues, including fat. This is associated with massive alterations of adipose tissue distribution, subcutaneous lipoatrophy, visceral adipose tissue hypertrophy and, in some cases, lipomatosis. Moreover, the endocrine properties of these patients are also perturbed and they show frequently abnormal low levels of the adipokines adiponectin and leptin [12], [13]. The mechanistic processes linking mtDNA depletion with specific alterations in adipose tissue mass, distribution and endocrine function are poorly known.

Recently, murine models of mitochondrial DNA depletion have been developed through targeted ablation of the mitochondrial thymidine kinase 2 gene (*Tk2*) or gene replacement with a non-functional form [14], [15]. TK2 is responsible for providing phosphorylated deoxythymidine within mitochondria in differentiated, non-proliferating, cells and tissues. Accordingly, TK2 is highly expressed in post-mitotic cells, in which the cytosolic form of thymidine kinase, TK1, is poorly expressed. By providing the deoxynucleotides required for mtDNA replication, TK2 activity plays an essential role in maintaining appropriate levels of mtDNA. Thus, null mutations of the *TK2* gene, such as those encoding the H121N mutant, lead to mtDNA depletion and multiple neuromuscular pathologies in patients [16], [17], [18], [19], [20]. Experimental inactivation of the *Tk2* gene in mice partially mimics these alterations [14], [15]. Thus, both targeted invalidation of the *Tk2* gene (Tk2-knockout) [15], and knockin of the enzymatically null H126N form of *Tk2* [14] cause early mortality in mice. It has been proposed that this is attributable to neuromuscular failure [14]. Moreover, the cellular morphology of the brain, spinal cord [14], and brown adipose tissue [15] is altered by either mutation. However, in either model, the impact of TK2 loss-of-function on mtDNA depletion in distinct tissues is highly variable. Profound mtDNA loss occurs in neural tissue but only very moderate depletion is evident in the liver or kidney.

TK2 loss-of-function rodent models may be useful tools for investigating the role of mtDNA depletion in adipose tissue. In the present study, we have counted for that aim with mice homozygous for knockin of the enzymatically null H126N *Tk2* mutant, homologous to the pathological H121N *TK2* mutation in humans. We have determined to what extent TK2- deficiency alters mtDNA levels in WAT and BAT, and the consequences on the size and function of adipose tissue depots.

## Materials and Methods

### Ethics statement

Mice were cared for and used in accordance with European Community Council Directive 86/609/EEC and approved by the Institutional Animal Care and Use Committee of the University of Barcelona (Approval no: DMAH 4100).

### Animals and tissue collection

Homozygous *Tk2* H126N knockin (Tk2^-/-^) mice were studied when thirteen-day-old, the age at which mice had not already developed over neuromuscular disease [14]. Tk2^-/-^ mice and wild-type (Tk2^+/+^) littermates were sacrificed, and interscapular brown (BAT) and anterior subcutaneous white (WAT) adipose tissue depots were dissected and frozen in liquid nitrogen. A small piece of tissue was separated, cut up into pieces, and stored at 4°C in fixation buffer (2% paraformaldehyde, 2.5% glutaraldehyde, 0.1 M phosphate buffer, pH 7.4) for subsequent transmission electron microscopy analyses. Newborn (1-day-old) Tk2^-/-^ and Tk2^+/+^ mice were also sacrificed to obtain BAT. For development studies, BAT and WAT from C57BL/6J wild-type mice were also collected**.**

### Preparation of tissue samples

For nucleic acid analyses, tissue samples (15–60 mg) were homogenized using a Polytron device (Ultra-Turrax, IKA Laboratory Equipment, Staufen, Germany), RNA was isolated using a single-column commercial kit (NucleoSpin, Macherey-Nagel GmbH and Co., Düren, Germany), and DNA was isolated using a phenol/chloroform extraction method. DNA and RNA were quantified spectrophotometrically (NanoDrop, Thermo Scientific, Waltham, MA, USA). For protein analyses, 15–40-mg tissue samples were homogenized in 300 µl of protein lyses buffer (20 mM Tris-HCl pH 7.4, 40 mM KCl, 2 mM EGTA, 5 mM PMSF) and protein was quantified using the Bradford method (Bio-Rad, Hercules, CA, USA). Tissue lipid content was measured gravimetrically after extraction, in accordance with previously described procedures [21].

### Evaluation of serum parameters

Glucose and lactate levels were measured in serum using Accutrend Technology (Roche Diagnostics, Basel, Switzerland). Adiponectin levels were determined by immunoassay using a commercial enzyme-linked immunosorbent assay kit (Linco Research, Saint Charles, MO, USA). Leptin,, interleukin-6, total plasminogen activator inhibitor type-1 and resistin were quantified in 20 µl of plasma using a multiplex system (Linco Research/Millipore, Saint Charles, MO, USA) and a Luminex100ISv2 equipment.

### Optical and transmission electron microscopy analyses

Fixed BAT and WAT samples were post-fixed in 1% osmium tetroxide and 0.8% FeCNK in phosphate buffer 0.1 M. After dehydration in a graded acetone series, tissue samples were embedded in Spurr resin. Ultrathin sections were obtained using an Ultracut UCT (Leica Microsystems GmbH, Wetzlar, Germany) and examined under an optical microscope. Samples were subsequently stained with uranyl acetate and lead citrate, and examined by transmission electron microscopy (JEOL 1010, Tokyo, Japan). White adipocyte area determination was performed using the ImageJ analysis software. For stereological analysis, the proportion of mitochondrial volume with respect to non-fat cell volume (Vol_mit_/Vol_non-fat cell_) was estimated using the volume density method [22]; surface density was calculated employing a vertical sections method [22], [23].

### mRNA expression analyses

Total RNA (0.5 µg) was transcribed into cDNA using MultiScribe reverse transcriptase and random-hexamer primers (TaqMan Reverse Transcription Reagents; Applied Biosystems, Foster City, CA, USA). For quantitative mRNA expression analyses, TaqMan reverse transcriptase-polymerase chain reaction (RT-PCR) was performed on the ABI PRISM 7700HT sequence detection system (Applied Biosystems). Reactions were performed in a final volume of 20 µl using TaqMan Universal PCR Master Mix, No-AmpErase UNG reagent, and the specific primer pair/probe sets (TaqMan Gene Expression Assays; Applied Biosystems) (see Table S1). On-Demand (Custom TaqMan Gene Expression Assays, Applied Biosystems) primers and probes were designed for the quantification of NADH dehydrogenase subunit 1 (ND1), cytochrome c oxidase subunit I (COXI) and cytochrome b (CYTB) mRNAs and mtDNA abundance. Sequences were 5′-GAG CCT CAA ACT CCA AAT ACT CAC T-3′ (forward), 5′-GAA CTG ATA AAA GGA TAA TAG CTA TGG TTA CTT CA-3′ (reverse) and 5′-CCG TAG CCC AAA CAA T-3′ (FAM-labeled probe) for ND1; 5′-GGT GCA ATT AAT TTT ATT ACC ACT ATT ATC AAC ATG A-3′ (forward), 5′-GGC TGT AAT AAG TAC GGA TCA GAC A-3′ (reverse) and 5′-CCC CAG CCA TAA CAC A-3′ (FAM-labeled probe) for COX1; and 5′-CCC AAC AGG ATT AAA CTC AGA TGC A-3′ (forward), 5′-CTA GGG TTA TGA GAA TTA AGA ATA TGA TTA GGA TAC CT-3′ (reverse) and 5′-TTC CAT TTC ACC CCT ACT ATA CA-3′ (FAM-labeled probe) for CYTB. Eukaryotic rRNA (18S; Hs99999901_s1) was used as reference control, and main results were confirmed by the use of the cyclophilin A transcript (Ppia; Mm02342430_g1) as an alternative housekeeping control.

### Assessment of relative mtDNA content

mtDNA was quantified by quantitative real-time PCR quantification of CYTB gene relative to nuclear DNA, determined by quantitative real-time PCR quantification of the Cebpa gene [24].

### Western blot and immunodetection

For Western blot analyses, cell lysates containing 15–30 µg protein were resolved by sodium dodecyl sulfate-poliacrylamide gel electrophoresis on 15% gels and transferred to polyvinylidene fluoride membranes (Immobilon, Millipore, Billerica, MA, USA). For immunodetection of OPA1, 7.5% poliacrylamide gels were used. Membranes were incubated with blocking buffer (5% dried milk/0.1% Tween-20/phosphate-buffered saline [PBS]) for 1 hour at room temperature, and incubated with the corresponding primary antibodies in 1% dried milk/0.1% Tween-20/PBS overnight at 4°C (see Table S2). Primary antibodies were immunodetected using polyclonal anti-mouse (Bio-Rad Laboratories, S.A., Spain) or anti-rabbit (Santa Cruz Biotechnology Inc., Santa Cruz, CA, USA) secondary horseradish peroxidase-conjugated antibodies. Enhanced chemiluminescence reagents were used to analyze the immunoreactive signals (Immobilon Western, Millipore, Billerica, MA, USA). All membranes were stained with Coomassie Brilliant Blue R-250 (Sigma Chemical Co., St. Louis, MO, USA) to verify equal protein loading. Immunoreactive signals were analyzed densitometrically using Multi Gauge v3.0 software (Fujifilm, Madrid, Spain).

### Mitochondrial respiratory chain activities and caspase-9 activity

Complex I+III activity was determined as the velocity of reduction of cytochrome c by the enzyme NADH cytochrome c reductase in the presence of NADH and rotenone. For this purpose, changes in absorbance at 550 nm were measured with and without rotenone [25]. Complex IV (cytochrome c oxidase) activity was measured using reduced cytochrome c as a substrate. Cytochrome c oxidation was monitored at 550 nm [26]. Complex V (ATP synthase) activity was determined using a commercial kit (Novagen, Merck KGaA, Darmstadt, Germany). Citrate synthase activity was used as a marker of mitochondrial content. This enzyme catalyzes the acetyl-coenzyme A oxaloacetate reaction that yields citrate and coenzyme A. This latter product was measured using 55′-dithiobis-(2-nitrobenzoic acid); the reaction was monitored at 412 nm [27]. Enzyme activity was expressed per milligram protein. Protein content was determined using the Coomassie Plus protein assay reagent (Pierce, Rockford, IL, USA).

Caspase-9 activity was measured in tissue extracts (5–20 µg protein/assay) using a luminescence-based system (Casapase-Glo 9 Assay, Promega, Madison, WI, USA). Data were expressed as relative luminescence units (RLU) per mg of tissue protein.

### Statistics

All values are given as means ± SEM. Mann-Whitney (nonparametric) test was used for statistical analyses. Grubbs' test was used to determine significant outliers (significance, alpha = 0.05). A *P* value of less than 0.05 was considered to be significant.

## Results

### WAT and BAT hypotrophy in Tk2^-/-^ mice

The weights of both, the anterior subcutaneous WAT and the interscapular BAT depots were significantly lower in Tk2^-/-^ mice than in wild-type littermates. When expressed relative to body weight, the difference remained but was statistically significant only for WAT. The total lipid content in WAT and BAT was significantly lower in Tk2^-/-^ mice than in Tk2^+/+^ mice, and accounted for most of the decrease in tissue weight in Tk2^-/-^ mice. Total protein content in WAT and BAT was not significantly different between Tk2^-/-^ and Tk2^+/+^ mice (Table 1).

**Table 1:** Body and tissue weights, protein and lipid content in 13-day-old *Tk2* H126N knockin mice (Tk2^-/-^).

	Tk2^+/+^	Tk2^-/-^
**Body weight (g)**	**6.3**±**0.2**	**5.5**±0.3**
**WAT**		
*** Weight (mg)***	**55.9**±**6.1**	**28.9**±5.5**
*** Weight body weight (mg/g)***	**9.0**±**0.8**	**5.5**±**1.0** *
* Protein content (mg)*	1.2±0.1	0.8±0.2
* Protein content (mg/g tissue)*	22.9±3.1	29.1±4.7
*** Lipid content (mg)***	**51.4**±**6.1**	**26.0**±3.8**
* Lipid content (mg/g tissue)*	919±87	899±92
**BAT**		
*** Weight (mg)***	**45.8**±**2.7**	**33.4**±2.2**
* Weight/body weight (mg/g)*	7.2±0.3	6.3±0.5
* Protein content (mg)*	1.7±0.1	1.6±0.2
* Protein content (mg/g tissue)*	38.6±5.3	47.7±7.4
*** Lipid content (mg)***	**26.6**±**2.3**	**16.4**±0.7**
* Lipid content (mg/g tissue)*	580±50	490±20

Values are means±SEM of 7–12 mice/group.

*P<0.05; **P<0.01

### Altered mitochondrial morphology in BAT from Tk2^-/-^ mice

**Figure 1:**
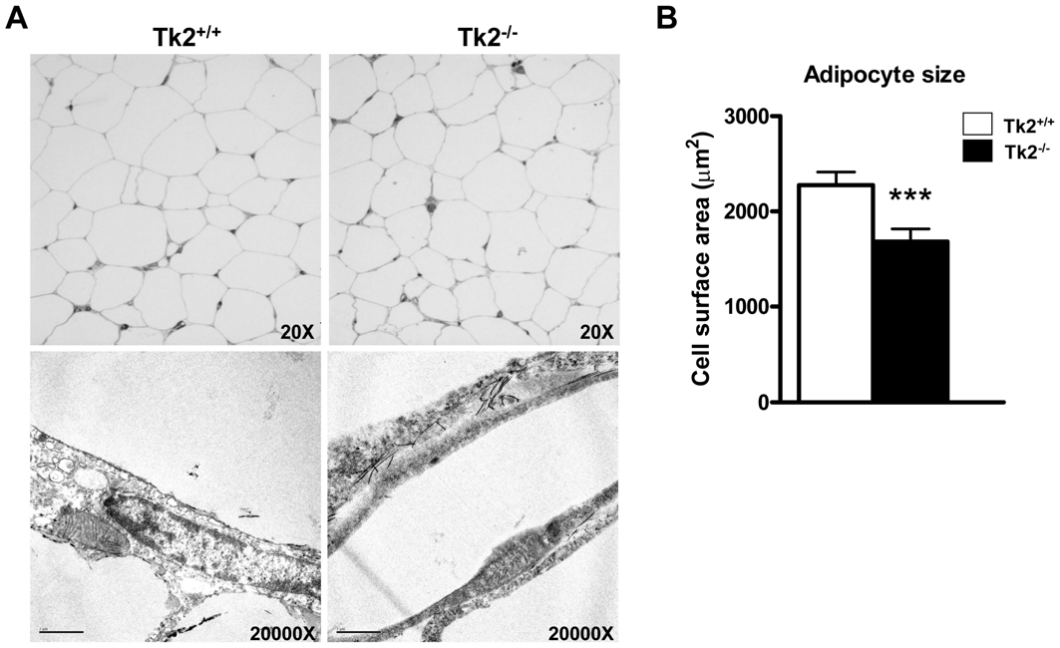
Morphology of WAT and mitochondria in white adipocytes from *Tk2* H126N knockin mice . A. Optical microscopy (top) and transmission electron microscopy analysis (bottom) of WAT from Tk2^+/+^ and Tk2^-/-^ mice. Scale bars: 1 µm (bottom panel). The image is representative of eight independent slides from three different animals for each genotype. B. White adipocyte size analysis from Tk2^+/+^ and Tk2^-/-^ mice. Bars are means ± SEM data expressed as cell surface area (µm2) (***P<0.001 for Tk2^+/+^ vs. Tk2^-/-^; 8 slides/group).

**Figure 2:**
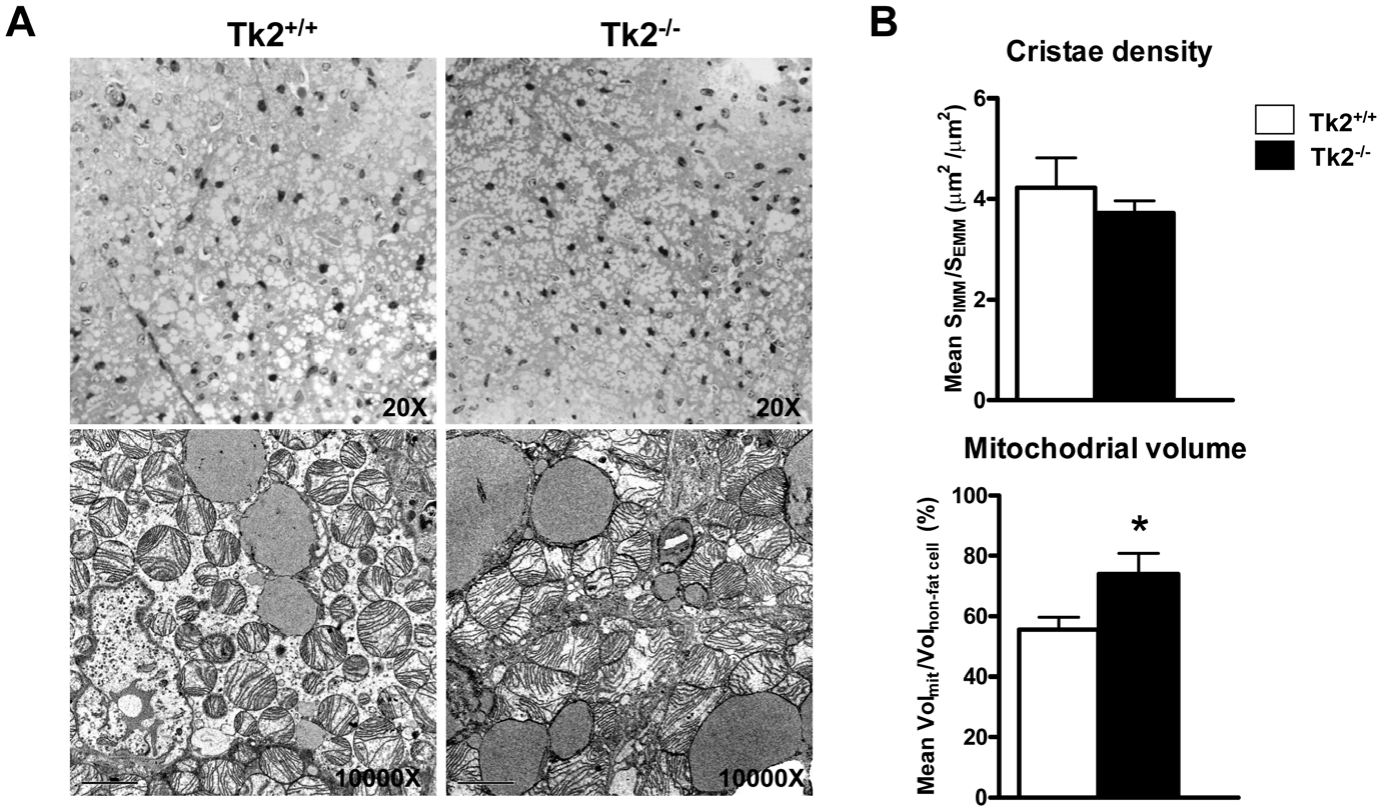
Morphology of BAT and mitochondria in brown adipocytes from *Tk2* H126N knockin mice. A. Optical microscopy (top) and transmission electron microscopy analysis (bottom) of BAT from Tk2^+/+^ and Tk2^-/-^ mice. Scale bars: 2 µm (bottom panel). The image is representative of six independent slides from three different animals for each genotype. B. Stereological analyses of mitochondrial cristae density and relative mitochondrial volume from Tk2^+/+^ and Tk2^-/-^ mice. Bars are means ± SEM data expressed as inner mitochondrial versus external mitochondrial membranes surface ratio (µm2/µm2), and mitochondrial volume versus non-fat cytoplasm volume (%) (*P<0.05 for Tk2^+/+^ vs. Tk2^-/-^; 8 slides/group), respectively.

Examination of WAT morphology by optical microscopy revealed no evidence of any gross alterations in adipocyte size or morphology (Fig. 1A, *top*). However, quantitative analysis of cell surface area revealed a moderate but highly significant reduction in adipocyte size in Tk2^-/-^ mice (Fig. 1B). Transmission electron microscopy (TEM) revealed no major changes in mitochondrial morphology in WAT from Tk2^-/-^ mice (Fig. 1A, *bottom*). Optical microscopic examination also revealed no alteration in the gross morphology of brown adipocytes in BAT from Tk2^-/-^ mice (Fig. 2A, *top*). However, examination of brown adipocytes by TEM revealed abnormal development of mitochondria in Tk2^-/-^ mice. Clumps of densely packed mitochondria were evident in the brown adipocytes of Tk2^-/-^ mice (Fig. 2A, *bottom*) and quantitative measurements revealed that mitochondria occupied a greater proportion of the cytoplasmatic volume (Fig. 2B, *bottom*). However, the mitochondria appeared to develop regularly, and contained well-formed cristae, as evidenced by surface ratio quantification data (Fig. 2B, *top*).

### mtDNA depletion and impaired expression of mtDNA-encoded transcripts in adipose tissues from Tk2^-/-^ mice

**Figure 3:**
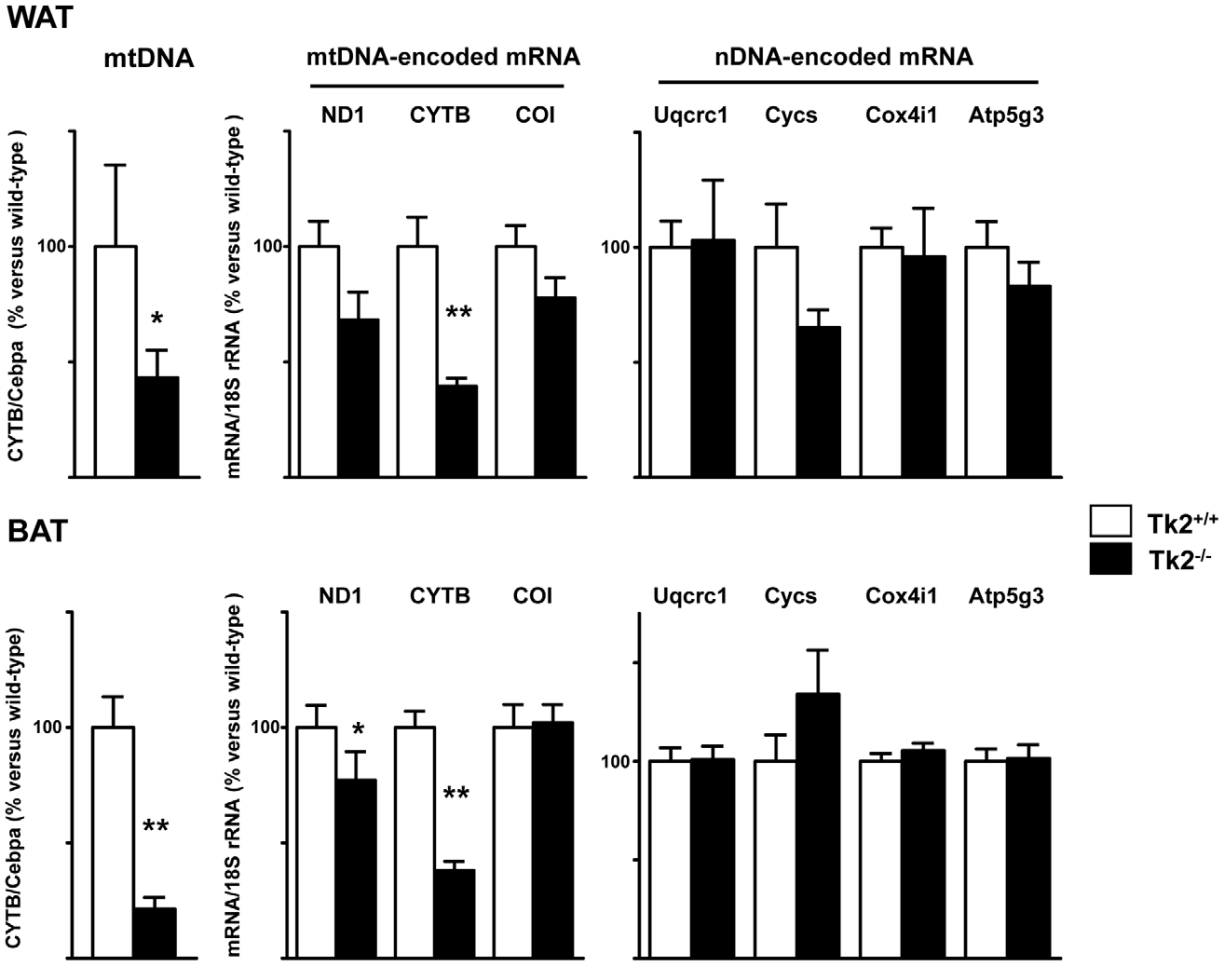
Mitochondrial DNA and OXPHOS transcript levels in WAT and BAT from *Tk2* H126N knockin mice. mtDNA relative abundance, and mtDNA- and nDNA-encoded transcript levels in WAT and BAT from Tk2^+/+^ and Tk2^-/-^ 13-day-old mice. Bars are means ± SEM data expressed as percentage values relative to wild-type controls (*P<0.05; **P<0.01 for Tk2^+/+^ vs. Tk2^-/-^; 7-12 mice/group). ND1, NADH dehydrogenase subunit 1; CYTB, cytochrome b; COI, cytochrome c oxidase subunit 1; Uqcrc1, ubiquinol-cytochrome c reductase core protein 1; Cycs, cytochrome c somatic; Cox4i1, cytochrome c oxidase subunit 4 isoform 1; Atp5g3, ATP synthase, H^+^ transporting, mitochondrial F_0_ complex, subunit c (subunit 9), isoform 3.

mtDNA levels were determined in WAT and BAT from Tk2^-/-^ mice and their Tk2^+/+^ littermates. BAT from Tk2^-/-^ mice showed a dramatic reduction in abundance of mtDNA, which was decreased to approximately 20% of the mtDNA levels in wild-type mice. A milder reduction in mtDNA content was observed in WAT form Tk2^-/-^ mice (Fig. 3). In BAT, the reduction in mtDNA levels was associated with a significant reduction in transcript levels of the mtDNA-encoded ND1 gene. A significant reduction in the mtDNA-encoded CYTB mRNA was observed in both WAT and BAT. In contrast, nDNA-encoded components of the OXPHOS system (Uqcrc1, Cycs, Cox4i1, and Atp5g3) were unaltered in both tissues (Fig. 3).

The levels of mtDNA in BAT could be analyzed in Tk2^-/-^ newborns, given that BAT, but not WAT, is already developed in neonatal mice. There were no significant changes in mtDNA relative abundance or mtDNA-encoded transcript (CYTB) levels in BAT from Tk2^-/-^ pups respect to controls (data not shown).

### Transcript levels of mitochondrial nDNA-encoded transcripts in WAT and BAT from Tk2^-/-^ mice

We further analyzed the transcript levels for up to eleven nuclear genes involved in distinct aspects of WAT and BAT mitochondrial functions. No statistically significant changes were found in WAT from Tk2^-/-^ mice compared to Tk2^+/+^ littermates in the expression levels of transcripts encoding components of the mtDNA expression machinery, of mitochondrial oxidative stress protection, or mitochondrial dynamics. In contrast, Tk2^-/-^ mice showed an altered gene expression in BAT of two genes involved in the oxidative stress and mitochondrial dynamics. *Sod2* mRNA levels were significantly decreased in Tk2^-/-^ compared to controls, whereas *Opa1* transcript levels appeared higher respect to wild-type littermates. *Ghitm* and *Immt* transcripts were also significantly reduced. No changes were found in BAT for the other genes analyzed (Table 2).

**Table 2:** Expression of the mRNA for nuclear genes encoding proteins involved in mitochondrial function in WAT and BAT from *Tk2* knockin mice.

	Tk2^-/-^ vs. Tk2^+/+^ (%)	P value
**WAT**		
mtDNA expression regulation		
* Transcription factor A, mitochondrial (Tfam)*	99 + 6	0.79
* Transcription factor B1, mitochondrial (Tfb1m)*	95 + 6	0.93
* Transcription factor B2, mitochondrial (Tfb2m)*	99 + 6	0.64
Mitochondrial ROS		
* Uncoupling protein-2 (Ucp2)*	102 + 20	0.61
* Superoxide dismutase 2 (Sod2)*	90 + 13	0.62
* Glutathione peroxidase4 (Gpx4)*	88 + 7	0.46
Mitochondrial dynamics		
* Optic atrophy 1 (Opa1)*	91 + 10	0.61
* Mitofusin 2 (Mfn2)*	119 + 18	0.63
**BAT**		
mtDNA expression regulation		
* Transcription factor A, mitochondrial (Tfam)*	82 + 7	0.34
* Transcription factor B1, mitochondrial (Tfb1m)*	87 + 4	0.15
* Transcription factor B2, mitochondrial (Tfb2m)*	92 + 7	0.64
* Mitochondrial translation initiation factor 2 (Mtif2)*	102 + 10	1.00
* Mitochondrial translation initiation factor 3 (Mtif3)*	84 + 14	0.66
Mitochondrial ROS		
* Uncoupling protein-2 (Ucp2)*	112 + 14	0.53
* Uncoupling protein-3 (Ucp3)*	100 + 6	0.70
*** Superoxide dismutase 2 (Sod2)***	67 + 4**	**0.004**
* Glutathione peroxidase 4 (Gpx4)*	83 + 6	0.09
Mitochondrial dynamics		
*** Optic atrophy 1 (Opa1)***	**138 + 11** *	**0.04**
* Mitofusin 2 (Mfn2)*	124 + 18	0.77
*** Growth hormone inducible transmembrane protein (Ghitm)***	**72 + 9** *	**0.04**
*** Mitochondrial inner membrane protein (Immt)***	**55 + 4** *	**0.02**

Values are expressed as means±SEM of the percent respect to control values that were set to 100 of 6–12 mice/group. P values for statistical comparisons are shown. Bold lettering is depicted when differences were statistically significant.

*P<0.05; **P<0.01

### Levels of mitochondrial proteins, respiratory complex activities and caspase-9 activity in adipose tissues from Tk2^-/-^ mice

**Figure 4:**
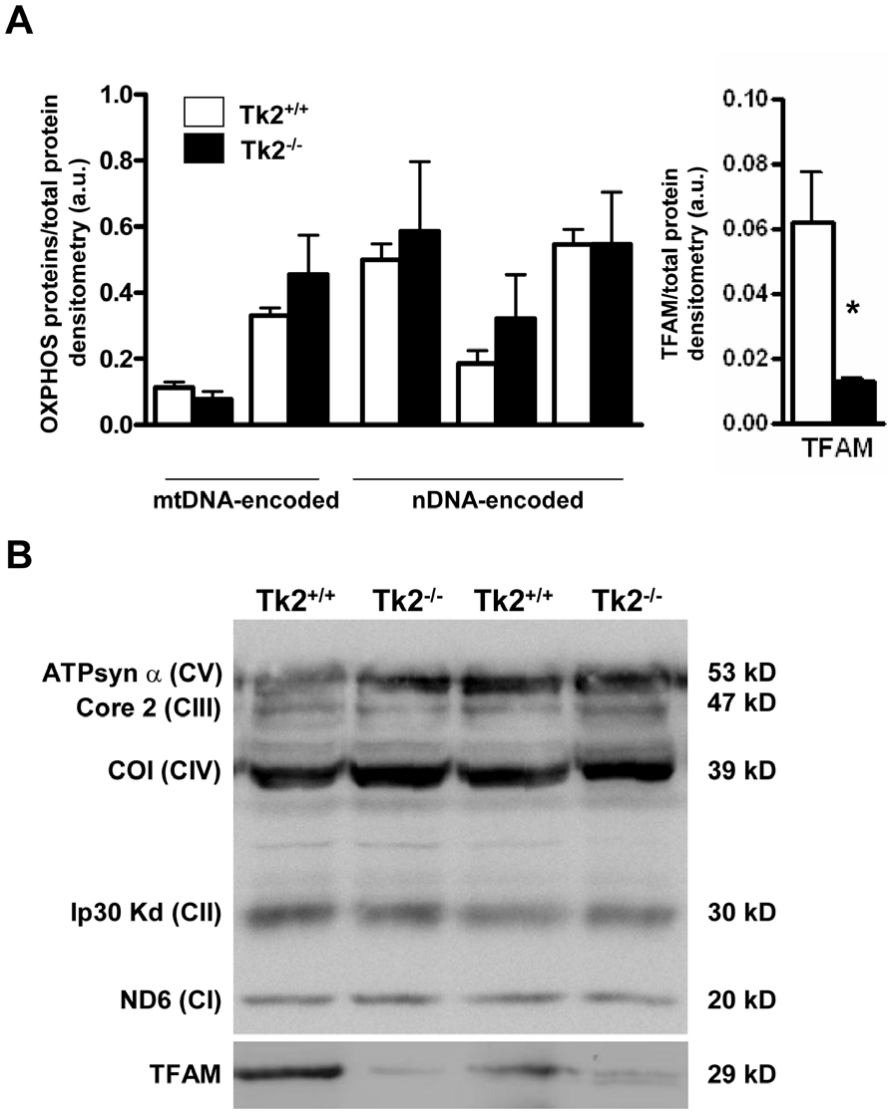
Immunoblot analyses of selected mitochondrial proteins in WAT from *Tk2* H126N knockin mice . A. Bars are means ± SEM of density of specific immunoblot signals normalized to total protein load (*P<0.05 for Tk2^+/+^ vs. Tk2^-/-^; 4-6 mice/group). B. Representative western blots of OXPHOS proteins and the mitochondrial transcription factor TFAM. ATPsyn α (CV), ATP synthase subunit α (Complex V); Core 2 (CIII), Core 2 subunit (Complex II); COI (CIV), cytochrome c oxidase subunit 1 (Complex IV); Ip30 kD (CII), 30 kD subunit (Complex II); ND6 (CI), NADH dehydrogenase subunit 6 (Complex I); TFAM, mitochondrial transcription factor A.

We next analyzed whether the mild mtDNA depletion and down-regulation of mtDNA-encoded transcripts in WAT from Tk2^-/-^ mice resulted in changes in the abundance of OXPHOS proteins. The results indicated no impairment in the expression of mtDNA- or nDNA-encoded OXPHOS protein components of distinct respiratory complexes in WAT (Fig. 4). In contrast, the levels of TFAM protein were dramatically reduced in WAT from Tk2^-/-^ mice (Fig. 4, *right*).

**Figure 5:**
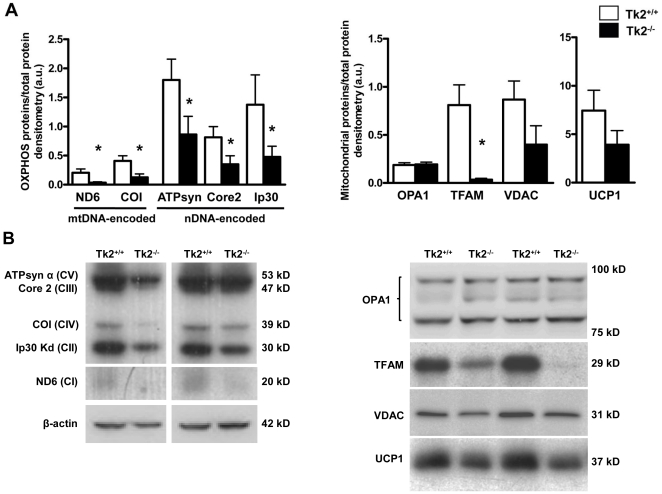
Immunoblot analyses of selected mitochondrial proteins in BAT from *Tk2* H126N knockin mice. A. Bars are means ± SEM of density of specific immunoblot signals normalized to total protein load (*P<0.05 for Tk2^+/+^ vs. Tk2^-/-^; 4-6 mice/group). B. Representative western blots. ATPsyn α (CV), ATP synthase subunit α (Complex V); Core 2 (CIII), Core 2 subunit (Complex II); COI (CIV), cytochrome c oxidase subunit 1 (Complex IV); Ip30 kD (CII), 30 kD subunit (Complex II); ND6 (CI), NADH dehydrogenase subunit 6 (Complex I); OPA1, Optic atrophy 1 protein; TFAM, mitochondrial transcription factor A; VDAC, voltage-dependent anion carrier; UCP1, uncoupling protein 1.

BAT from Tk2^-/-^ mice showed a significant reduction in the protein levels of the mtDNA-encoded proteins ND6 and COI. Nuclear encoded OXPHOS protein levels were also reduced (Fig. 5, *left*). A dramatic reduction of TFAM to nearly undetectable levels was observed in BAT from Tk2^-/-^ mice. Although there was a trend towards reduced levels of UCP1 and VDAC proteins, these differences did not reach statistical significance (Fig. 5, *right*). No significant changes were found for OPA1 fusion protein isoforms between Tk2^-/-^ and wild-type mice (Fig. 5, *right*). These data indicate that mtDNA depletion in Tk2^-/-^ mice was associated with a specific impairment in the expression of OXPHOS components in BAT.

**Figure 6:**
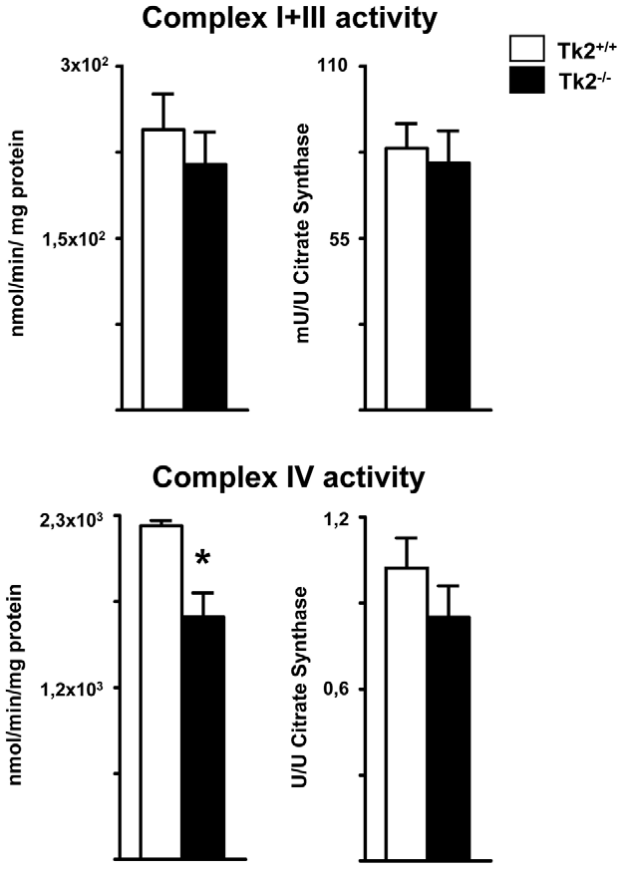
Mitochondrial respiratory complex activities in BAT from *Tk2* H126N knockin mice. Mitochondrial respiratory complex I+III and complex IV (cytochrome c oxidase) activities in BAT from Tk2^+/+^ and Tk2^-/-^ mice. The activity of each complex is expressed as nanomoles of final product per minute (1 Unit) per milligram protein, and as mUnits (or Units) of complex activity/Units of citrate synthase activity. Bars are means ± SEM (*P<0.05 for Tk2^+/+^ vs. Tk2^-/-^; 5–10 mice/group).

In order to determine whether these changes had consequences for mitochondrial OXPHOS function, we analyzed the enzymatic activity of mitochondrial respiratory complexes. Whereas there were no significant differences in complex I+III activity (Fig. 6, *top*), there was a significant reduction in complex IV (cytochrome c oxidase) activity in BAT from Tk2^-/-^ mice compared to controls (Fig. 6, *bottom*). When complex IV activity was expressed as relative to citrate synthase activity, an index of mitochondrial mass, activity levels remained lower, but the difference was not statistically significant (Fig. 6, *bottom, right*). Assessment of complex V (ATP synthase) activity indicated very low enzymatic activity (in the 5–6 nmol/min/mg protein range) in BAT from wild-type mice, in accordance with previous reports [28], and no significant differences were found in Tk2^-/-^ mice (data not shown).

In order to explore whether alterations in mitochondrial morphology and complex IV activity in BAT may result in alterations in mitochondria-driven apoptotic activation, we measured caspase-9 activity. Results indicated no significant differences between Tk2^+/+^ (12.6±1.2 RLU/mg protein) and Tk2^-/-^ mice (13.6±1.1 RLU/mg protein), consistently with the lack of morphological indications of apoptosis in the microscopy analysis of tissues.

### Leptin and resistin depletion in serum from Tk2^-/-^ mice

The analysis of serum adipokines revealed a strong reduction in the levels of leptin and resistin in Tk2^-/-^ mice compared to control littermates. These changes occurred in the absence of significant alterations in other circulating parameters, such as serum glucose, or serum lactate. No changes were found in other adipokines, such as adiponectin, or pro-inflammatory cytokines, such as interleukin-6 and PAI-1α (Table 3).

**Table 3:** Serum parameters in 13-day-old *Tk2* H126N knockin mice (Tk2^-/-^).

	Tk2^+/+^	Tk2^-/-^
Glucose (mg/dl)	139.80±20.30	123.30±17.37
Lactate (mM)	4.78±1.41	4.65±0.93
Adiponectin (mg/ml)	6.55±0.46	5.34±0.24
Interleukin-6 (pg/ml)	17.77±3.65	11.10±2.87
**Leptin (ng/ml)**	**4.11**±**0.68**	**0.91**±0.17**
PAI-1α (ng/ml)	2.36±0.47	2.20±0.76
**Resistin (ng/ml)**	**5.24**±**0.74**	**2.65**±**0.57** *

Values are means±SEM of 7–12 mice/group.

*P<0.05; **P<0.01

### Impaired expression of adipokine and thermogenesis-related genes in WAT and BAT from Tk2-/- mice

**Figure 7:**
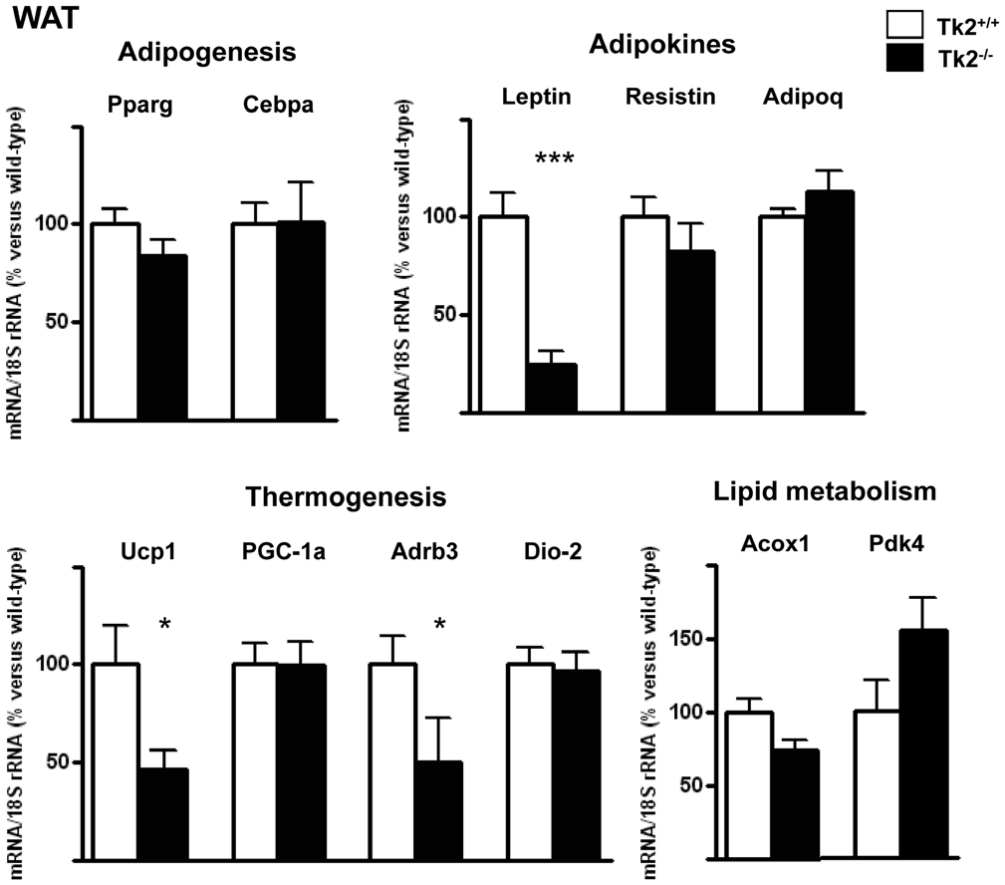
Expression of nuclear genes encoding proteins involved in adipogenesis, thermogenesis, and lipid metabolism in WAT from TK2-deficient mice. Bars are means ± SEM of the percent respect to control values that were set to 100 (*P<0.05; ***P<0.001 for Tk2^+/+^ vs. Tk2^-/-^; 6–12 mice/group). Pparg, peroxisome proliferator activated receptor-γ; Cebpa, CCAAT/enhancer-binding protein-α; Ucp1, uncoupling protein 1; PGC-1a, PPARγ coactivator-1α; Adrb3, β3-adrenergic receptor; Dio-2, 5′-deiodinase-2; Adipoq, adiponectin; Acox1, acyl CoA oxidase; Pdk4, pyruvate dehydrogenase-kinase 4.

**Figure 8:**
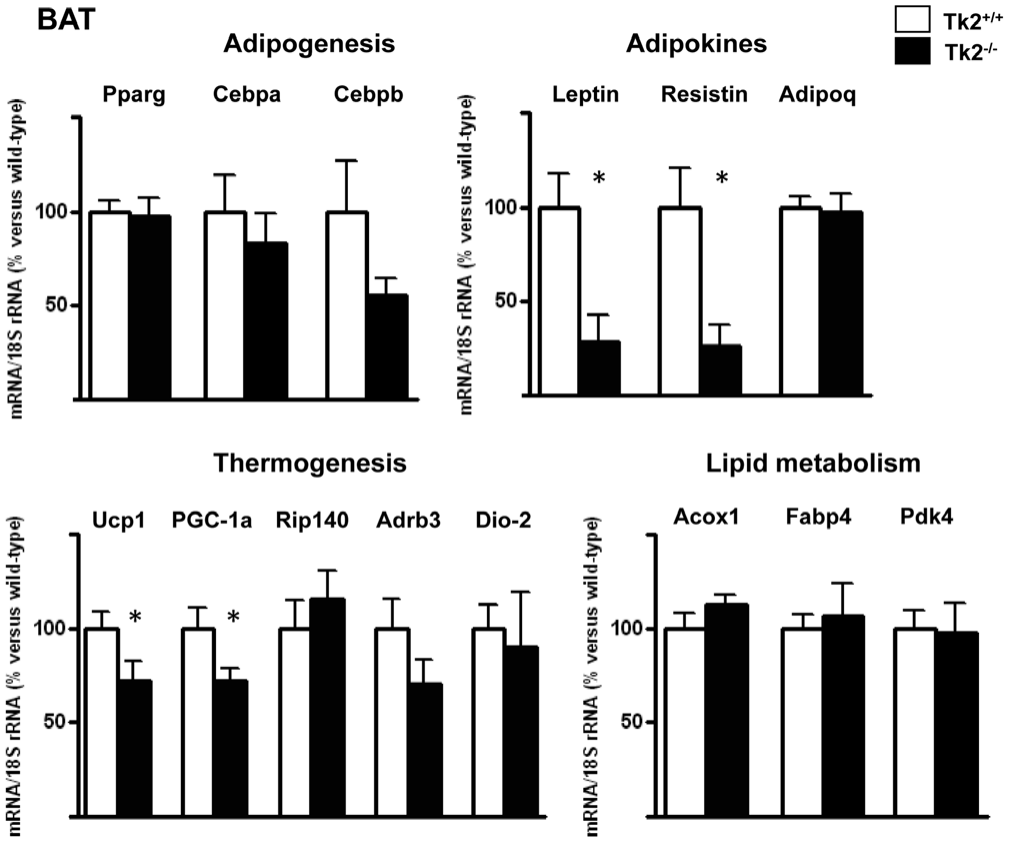
Expression of nuclear genes encoding proteins involved in adipogenesis, thermogenesis, and lipid metabolism in BAT from TK2-deficient mice. Bars are means ± SEM of the percent respect to control values that were set to 100 (*P<0.05 for Tk2^+/+^ vs. Tk2^-/-^; 8–12 mice/group). Pparg, peroxisome proliferator activated receptor-γ; Fabp4, fatty acid binding protein-4; Cebpa, CCAAT/enhancer-binding protein-α; Cebpb, CCAAT/enhancer-binding protein-β; Adipoq, adiponectin; Ucp1, uncoupling protein 1; PGC-1a, PPARγ coactivator-1α; Rip140, receptor interacting protein 140 kD; Adrb3, β3-adrenergic receptor; Dio-2, 5′-deiodinase-2; Acox1, acyl CoA oxidase; Pdk4, pyruvate dehydrogenase-kinase 4.

Finally, we analyzed the transcript levels for up to 14 nuclear genes involved in distinct aspects of WAT and BAT metabolism and physiological functions. No statistically significant changes were found in WAT or BAT from Tk2^-/-^ mice respect to Tk2^+/+^ littermates for the expression of transcripts encoding components of overall adipogenesis (Pparg, Cebpa, Cebpb) and of lipid metabolism (Acox1, Fabp4, Pdk4) (Fig. 7, Fig. 8). In contrast, the expression of two genes encoding proteins specifically involved in thermogenesis, the uncoupling protein-1 (Ucp1) and the β3 adrenergic receptor (Adrb3) were significantly down-regulated in WAT from Tk2^-/-^ mice (Fig. 7). Moreover, Ucp1 and PGC-1a transcripts were also down-regulated in BAT from Tk2^-/-^ mice (Fig. 8). An examination of the expression of Ucp1 and PGC-1a in BAT from 1-day-old newborn mice revealed no significant changes in Tk2^-/-^ pups (Ucp1, 96%±11% vs. wild-type; PGC-1a, 128%±9% vs. wild-type). Leptin mRNA levels showed a dramatic reduction in WAT and BAT from Tk2^-/-^ mice, whereas resistin transcript levels were only reduced in BAT. No statistically significant differences were found in WAT or BAT for adiponectin mRNA expression levels between Tk2^-/-^ mice and wild-type littermates (Fig. 7, Fig. 8).

### Developmental regulation of Tk2 gene expression and mtDNA levels in BAT and WAT

**Figure 9:**
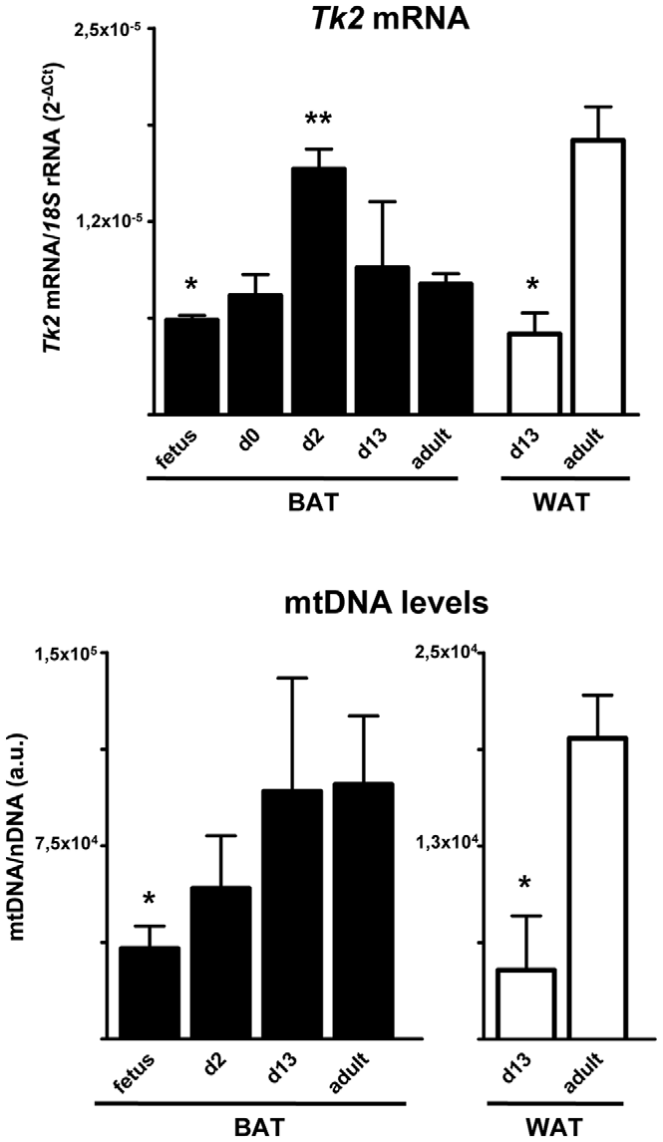
*Tk2* mRNA expression and mtDNA levels in BAT and WAT during mouse development. Bars are means ± SEM (*P<0.05; **P<0.01 for differences between adult BAT and BAT at different development stages, and between 13-day-old WAT and adult WAT; 3–8 mice/group).

We determined the profile of changes in *Tk2* mRNA expression and mtDNA abundance in adipose tissues throughout development of wild-type mice. *Tk2* mRNA levels were low in BAT from fetuses at term and it increased progressively in neonates, reaching adult levels shortly after birth. In 13 day-old mice, *Tk2* mRNA levels in BAT were already as high as in adults (Fig. 9, *top*). However, in WAT from 13 day-old pups *Tk2* mRNA levels had not yet reached adult levels. These results were paralleled by developmental changes in mtDNA levels: mtDNA abundance was low in fetal BAT but had attained adult levels 13 days after birth. In contrast, mtDNA levels in WAT were lower than in adult WAT (Fig. 9, *bottom*).

## Discussion

In the present study, Tk2^-/-^ mice were used as a model to investigate the role of mtDNA depletion in adipose tissues and to establish the specific alterations in WAT and BAT that take place as a consequence of TK2 loss-of-function and mtDNA depletion. TK2 deficiency resulted in a moderate reduction in the amounts of WAT and BAT in 13-day-old mice.

The extent of mtDNA depletion in BAT from 13-day-old Tk2^-/-^ mice was profound, about an 80–90%. This value was within the range of that in other highly affected tissues, such as brain and spinal cord, and was stronger than that in tissues such as liver and kidney, which showed about 30–40% depletion in this rodent model [14]. Remarkably, no such depletion was found in BAT from 1-day-old Tk2^-/-^ neonates, indicating that mtDNA depletion in BAT develops during the postnatal period. This observation is consistent with the profile of *Tk2* expression during BAT development, which showed a marked postnatal induction. During the late fetal period, BAT develops and differentiates progressively as a result of increased proliferation and differentiation of brown adipocytes [29]. However, after birth, proliferative processes are expected to be much reduced and BAT becomes an essentially post-mitotic tissue. The increase in *Tk2* gene expression in postnatal BAT is likely to be associated with the post-mitotic status of brown adipocytes after birth, and could explain why BAT mtDNA replication becomes strongly dependent on TK2 activity basically in the postnatal period.

The milder reduction in mtDNA levels in WAT may be related to the delayed development of WAT compared to BAT in rodents. It is known that there is almost no WAT in rodents at birth and, two weeks later, WAT has only begun to develop [30]. This is consistent by the already low levels of mtDNA and *Tk2* gene expression observed in WAT from 2-week-old mice compared to that in adults. This could also explain a minor dependency of WAT on TK2 activity in maintaining mtDNA levels during this stage of development, when WAT is still proliferatively active.

The profound depletion of mtDNA in BAT resulted in a reduction in the levels of mtDNA-encoded transcripts, as well as in the corresponding mtDNA-encoded OXPHOS proteins. The observed reduction in nDNA-encoded OXPHOS proteins, despite unaltered transcript levels, might be secondary to the original reduction in mtDNA-encoded proteins. Accordingly, impaired synthesis of mtDNA-encoded proteins may alter the stoichiometry of the subunit composition of OXPHOS complexes, resulting in concomitantly lower levels of the nDNA-encoded OXPHOS subunits. The net consequence of these alterations for mitochondrial respiratory complexes was a significant, although quantitatively moderate, reduction in complex IV activity.

Notably, TFAM protein levels, but not *Tfam* transcript levels, were dramatically down-regulated in BAT. This may reflect the known role of TFAM as an mtDNA-coating protein [31]. It has been reported that mtDNA depletion in patients is accompanied by low levels of TFAM without a decrease in its mRNA levels [32]. It is likely, therefore, that TFAM reduction was indirectly caused by the reduction in mtDNA levels elicited by TK2 loss-of-function.

We have shown that mtDNA depletion is not associated with massive alterations in white or brown adipocyte development or cell morphology; instead, only a quantitative reduction in tissue mass, mostly attributable to lower fat accretion. However, electron microscopy revealed alterations in mitochondrial distribution in brown adipocytes from Tk2^-/-^ mice, essentially the presence of clumps of highly packaged mitochondria in the cell cytoplasm associated with an increase in mitochondrial volume within the cell. This morphological abnormality is reminiscent of the accumulation of aberrant mitochondria that appears in the muscle of patients with some mtDNA diseases that lead to ragged-red fibers [33]; or to the mitochondrial proliferation reported in the adipose tissue from HIV-1 patients with lipodystrophy and mtDNA depletion [34], [35], [36]. The impaired expression of mitofilin (*Immt*) and MICS1 (*Ghitm*), marker genes for cristae organization and remodeling, is compatible with altered mitochondrial morphology despite quantitative assessment of cristae density did not evidence a significant change. However, the expression patterns of components of the mitochondrial proliferation, such as VDAC, or of the mitochondrial fusion machinery, such as OPA1, did not suggest massive enhancement of mitochondrial proliferation or disruption of mitochondrial dynamics as a consequence of mtDNA depletion in BAT. Further research will be required to establish the relationship between mitochondrial morphological alterations and the mild alteration in mitochondrial oxidative function in BAT from Tk2^-/-^ mice.

When we explored the possibility that TK2 loss-of-function and mtDNA depletion causes changes in specific adipocyte functions two major alterations were observed. First, we found abnormally down-regulation of marker genes of brown fat thermogenesis in both BAT and WAT depots. This observation may be consistent with the hypothermia previously reported in a similar *Tk2*-null mouse model [15]. However, as such temperature decrease occurs at an age very close to mortality, it is unclear whether hypothermia may be a primary event or simply a consequence of ongoing systemic breakdown attributable to neuromuscular disease. It may be that, through unknown mechanisms, the reduced mtDNA levels specific impairs the expression of thermogenesis-related genes in adipocytes. However, in light of the multi-systemic mtDNA depletion that occurs in Tk2^-/-^ mice [14], other possibilities should also be considered. For instance, BAT thermogenic activity is known to be under the control of sympathetic nervous system action on BAT [37]. Considering that nervous tissue is among the most affected tissues in Tk2^-/-^ mice, the possibility that the reduction of thermogenic activity in BAT was not a direct consequence of mtDNA depletion in brown adipocytes, but was rather an indirect consequence of altered sympathetic nervous system activity cannot be excluded. This phenomenon, if confirmed, could be relevant to understanding the impact of reduced mtDNA levels in humans, considering the recent evidence for active BAT under the control of sympathetic nervous system activity in adult humans [38], [39].

A relevant finding in the present study was that mtDNA depletion associated with TK2 deficiency causes a profound hypoleptinemia in mice. This could not be simply attributed to reduced amounts of adipose tissue, as there was a dramatic specific impairment in leptin gene expression both in BAT and WAT. Although in adult mice WAT instead of BAT is the main source of leptin, in very young rodents BAT seems to contribute to leptin synthesis and release [40], [41]. The reasons by which mtDNA depletion and mild respiratory activity reduction leads to such specific impairment in leptin gene expression is unclear, as the expression of known transcriptional activators of the leptin gene, such as C/EBPα and PPARγ [42], [43], are not modified in adipose tissue from Tk2^-/-^ mice. It has been reported that the gene expression for other adipokines, such as adiponectin, is strongly influenced by reactive oxygen species-dependent signaling originating in mitochondria [44]. Further research will be required to determine the mechanisms for altered mitochondrial retrograde signaling in adipose tissue from Tk2^-/-^ mice in response to mtDNA depletion. In is indeed true that hypoleptinemia may deleteriously affect overall energy metabolism but the pathophysiological consequences of low leptin levels require further research, especially considering the specificities of leptin secretion and action in mice at early stages of postnatal development [40], [41], [45]. Similar considerations may be raised for resistin; in this case, expression of resistin is known to be similar in BAT and WAT depots from young rodents [46], and impaired levels of resistin in serum are likely to be due to the strong reduction of resistin gene expression in BAT in response to mtDNA depletion.

In summary, this study constitutes the first report on the role of mtDNA depletion on adipose tissues in an *in vivo* experimental model. We established that very low levels of mtDNA cause mild impairment in BAT development, demonstrating the existence of cellular regulatory mechanisms that mitigate the potentially massive impairment in mitochondrial function that might otherwise accompany profound mtDNA depletion. Because our analyses required the use of very young mice (an intrinsic limitation of the current mouse model) more refined research models, such as conditional *Tk2*-null mice, will be required to determine the effects of TK2 deficiency in mtDNA in BAT and WAT at more advanced stages of development, and to establish whether the preferential impact of TK2 deficiency on mtDNA depletion in BAT compared to WAT is due to the characteristic temporal development profiles of these tissues in mice or to different intrinsic sensitivities of these two types of adipose tissues to TK2 deficiency and mtDNA alterations. Additional studies employing rodent models of mtDNA deficiency will also be helpful in determining the precise role of mtDNA depletion in the lipodystrophy syndrome that manifests in some HIV-infected patients undergoing antiretroviral treatment, a condition in which WAT mtDNA levels are abnormally reduced. Importantly, the existence of cellular mechanisms capable of alleviating the consequences of profound mtDNA depletion in adipose tissue, evidenced here, is also a subject that warrants further research, as the molecular components of these mechanisms constitute potential therapeutic targets for ameliorating disturbances in mtDNA depletion syndromes in humans. Finally, the present study evidences the existence of powerful mechanisms of mitochondrial retrograde signaling in adipose tissue, by which mild alterations in mitochondrial activity lead to profound abnormalities in gene expression for adipokines and subsequently on blood adipokine levels. This observation highlights that mitochondrial impairment in adipose tissue may expand to systemic metabolism when, as observed here, the nuclear gene expression affected by altered mitochondrial signaling corresponds to components of the endocrine function of the adipose tissue. This may be also relevant to explain phenomena such as hypoleptinemia often observed in HIV-1-infected patients with lipodystrophy [47] in association with a significant, but quantitatively moderate, depletion of mtDNA levels in adipose tissue.

## Supporting Information

Table S1 TaqMan Gene Expression Assays.
(DOC)

Table S2 Primary antibodies.
(DOC)
