# Supplementary material for: Thymidine Kinase 2 Deficiency-Induced Mitochondrial DNA Depletion Causes Abnormal Development of Adipose Tissues and Adipokine Levels in Mice
Source: PLoS One. 2011 Dec 27;6(12):e29691. doi: 10.1371/journal.pone.0029691 (PMC3246498; doi:10.1371/journal.pone.0029691)
Supplement: Table S1 — TaqMan Gene Expression Assays. (DOC) [file pone.0029691.s001.doc]

| **Gene Name** | **Gene Symbol** | **Assay ID** |
| --- | --- | --- |
|  |  |  |
| Mitochondrial transcription factor A | Tfam | Mm00447485_m1 |
| Mitochondrial transcription factor B1 | Tfb1m | Mm00524825_m1 |
| Mitochondrial transcription factor B2 | Tfb2m | Mm01620397_s1 |
| Mitochondrial translation initiation factor 2 | Mtif2 | Mm00505356_m1 |
| Mitochondrial translation initiation factor 3 | Mtif3 | Mm00512893_m1 |
| Uncoupling protein 1 | Ucp1 | Mm00494069_m1 |
| Uncoupling protein 2 | Ucp2 | Mm00495907_g1 |
| Uncoupling protein 3 | Ucp3 | Mm00494074_m1 |
| Superoxide dismutase 2 | Sod2 | Mm00449726_m1 |
| Receptor interacting protein 140kD | Rip140 | Mm01343436_m1 |
| 5’-deiodinase-2 | Dio-2 | Mm00515664_m1 |
| Fatty acid binding protein-4 | Fabp4 | Mm00445880_m1 |
| Peroxisome proliferator activated receptor-γ | Pparg | Mm00440945_m1 |
| PPARγ coactivator-1α | Ppargc1a | Mm00447183_m1 |
| β3-adrenergic receptor | Adrb3 | Mm00442669_m1 |
| Adiponectin | Adipoq | Mm00456425_m1 |
| Acyl CoA oxidase | Acox1 | Mm00443579_m1 |
| CCAAT/enhancer-binding protein-α | Cebpa | Mm00514283_s1 |
| CCAAT/enhancer-binding protein-β | Cebpb | Mm00843434_s1 |
| Pyruvate dehydrogenase-kinase 4 | Pdk4 | Mm00443325_m1 |
| Optic atrophy 1 | Opa1 | Mm00453879_m1 |
| Mitofusin 2 | Mfn2 | Mm00500120_m1 |
| Thymidine kinase 2 | Tk2 | Mm00445175_m1 |
| Cytochrome b-c1 complex subunit 1 | Uqcrc1 | Mm00445911_m1 |
| Cytochrome c somatic | Cycs | Mm01621044_g1 |
| Cytochrome c oxidase subunit IV, isoform 1 | Cox4i1 | Mm00438289_g1 |
| ATP synthase H^+^ transporting F_0_ complex subunit c | Atp5g3 | Mm00558162_m1 |
| Leptin | Lep | Mm00434759_m1 |
| Resistin | Retn | Mm00445641_m1 |
| Mics1 | Ghitm | Mm00504440_m1 |
| Mitofilin | Immt | Mm00518636_m1 |

**Supplementary Table 1: TaqMan Gene Expression Assays**
