# Supplementary material for: Thymidine Kinase 2 Deficiency-Induced Mitochondrial DNA Depletion Causes Abnormal Development of Adipose Tissues and Adipokine Levels in Mice
Source: PLoS One. 2011 Dec 27;6(12):e29691. doi: 10.1371/journal.pone.0029691 (PMC3246498; doi:10.1371/journal.pone.0029691)
Supplement: Table S2 — Primary antibodies. (DOC) [file pone.0029691.s002.doc]

| **Antibody** | **Conc./Dilution** | **Source** |
| --- | --- | --- |
|  |  |  |
| Rodent Total OXPHOS Cocktail | 6 µg/ml | MitoSciences, Eugene, OR, USA |
| TFAM monoclonal | 1:200 | Kind gift from M. Rojo (INSERM, France) |
| OPA1 polyclonal | 2 µg/ml | Abcam, Cambridge, UK |
| UCP1 polyclonal | 1:200 | Kind gift from E. Rial (CIB-CSIC, Madrid, Spain) |
| VDAC monoclonal | 1 µg/ml | Calbiochem, EMD Biosciences Inc., La Jolla, CA, USA |
| β-actin monoclonal | 1:10000 | Sigma-Aldrich, St. Louis, MO, USA |

**Supplementary Table 2: Primary antibodies**
